# Supplementary material for: Allele‐specific silencing therapy for Dynamin 2‐related dominant centronuclear myopathy
Source: EMBO Mol Med. 2017 Dec 15;10(2):239–53. doi: 10.15252/emmm.201707988 (PMC5801507; doi:10.15252/emmm.201707988)
Supplement: Supplementary file 1 — Appendix [file EMMM-10-239-s001.pdf]

Appendix

Allele-specific silencing therapy for dynamin 2-related dominant centronuclear myopathy

Delphine Trochet[1], Bernard Prudhon[1], Maud Beuvin[1], Cécile Peccate[1], Stéphanie Lorain[1], Laura Julien, Sofia Benkhelifa-Ziyyat[1], Aymen Rabai[3,] Kamel Mamchaoui[1], Arnaud Ferry[1], Jocelyn Laporte, Pascale Guicheney[2], Stéphane Vassilopoulos[1], Marc Bitoun[1]*

Table of content

Appendix Fig. S1. RT-PCR assay for identification of allele-specificity in MEF.

Appendix Fig. S2. Effect of the allele-specific si9 and si10 sequences on *Dnm1*, *Dnm2* and *Dnm3* expression.

Appendix Fig. S3. si9 and si10 are allele-specific siRNAs in Mouse Myoblast

Appendix Fig. S4. Expression of WT and mutant *Dnm2* mRNAs in AAV-transduced mouse muscles.

Appendix Fig. S5. Effect of 1 month Sh9 treatment in young mice.

Appendix Fig. S6. Quantification of viral genome per nanogram.

Appendix Fig. S7. RT-PCR assay for identification of allele-specific siRNAs in human cells.

Appendix Fig. S8. Allele-specific silencing induced by si9 in human fibroblasts at high doses.

Appendix Fig.S9. In silico off-targets prediction for human and mouse si9 RNA sequences

Appendix Fig. S10. Uncropped gels and western blots from main Figures.

Appendix Fig. S11. Uncropped gels from Appendix Figures.

Appendix Table S1. siRNA molecules targeting *Dnm2* mutant mRNA in this study.

Appendix Table S2. Primers used in this study

Appendix Table S3. Individual P-values for main Figures

Appendix Table S4. Individual P-values for Appendix Figures

Appendix Figure S1

A

```
GGTGGTCAAGCTGAAAGAGCCCTGTCTGAAATGCGTGGACCTGGTTATCCAGGAGCTAATCAGTACAGTT
AGGCAGTGCACCAGCAAGCTGAGTTCCTACCCCCGGCTGCGAGAGGAGACCGAGCGAATTGTCACCACCT
ACATCAGGGAGCGAGAAGGGAGAACCAAGGACCAGATTCTTCTTCTGATTGACATTGAGCAGTCGTACAT
CAACACAAACCATGAAGACTTCATTGGATTTGCCAATGCCCAGCAGAGGAGCACGCAGCTGAACAAGAAG
AGGGCCATACCCAATCAGGGGGAGATCTTGGTGATCCGCAGGGGCTGGTTGACCATCAACAATATCAGCT
TGATGAAGGGTGGCTCCAAGGAGTACTGGTTCGTGCTGACAGC
```

B

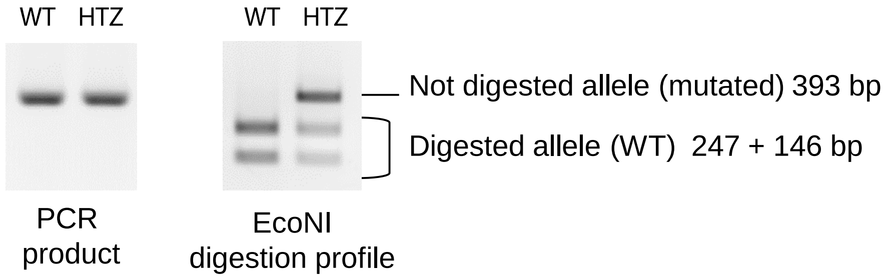

Appendix Figure S1. RT-PCR assay for identification of allele-specificity in MEF.

(**A**) Nucleotide sequence of the mouse Dnm2 RT-PCR product in the region of the mutation. Primers are underlined, EcoNI site is in bold and position of the mutation (A>T) in red. Presence of the mutation abolishes the recognition site for EcoNI.

(**B**) Agarose gel electrophoresis of non-digested PCR amplicons (left) or after EcoNI digestion (right) for WT and HTZ MEFs. EcoNI digests only RT-PCR product amplified from the WT allele allowing discrimination between WT and mutated alleles and quantification of signal associated with both alleles. The associated-signal for the WT allele is obtained by the sum of the signal for the two bands. WT sample is used as internal control showing total digestion under these conditions. Length of each band in base-pairs (bp) is indicated on the right.

Appendix Figure S2

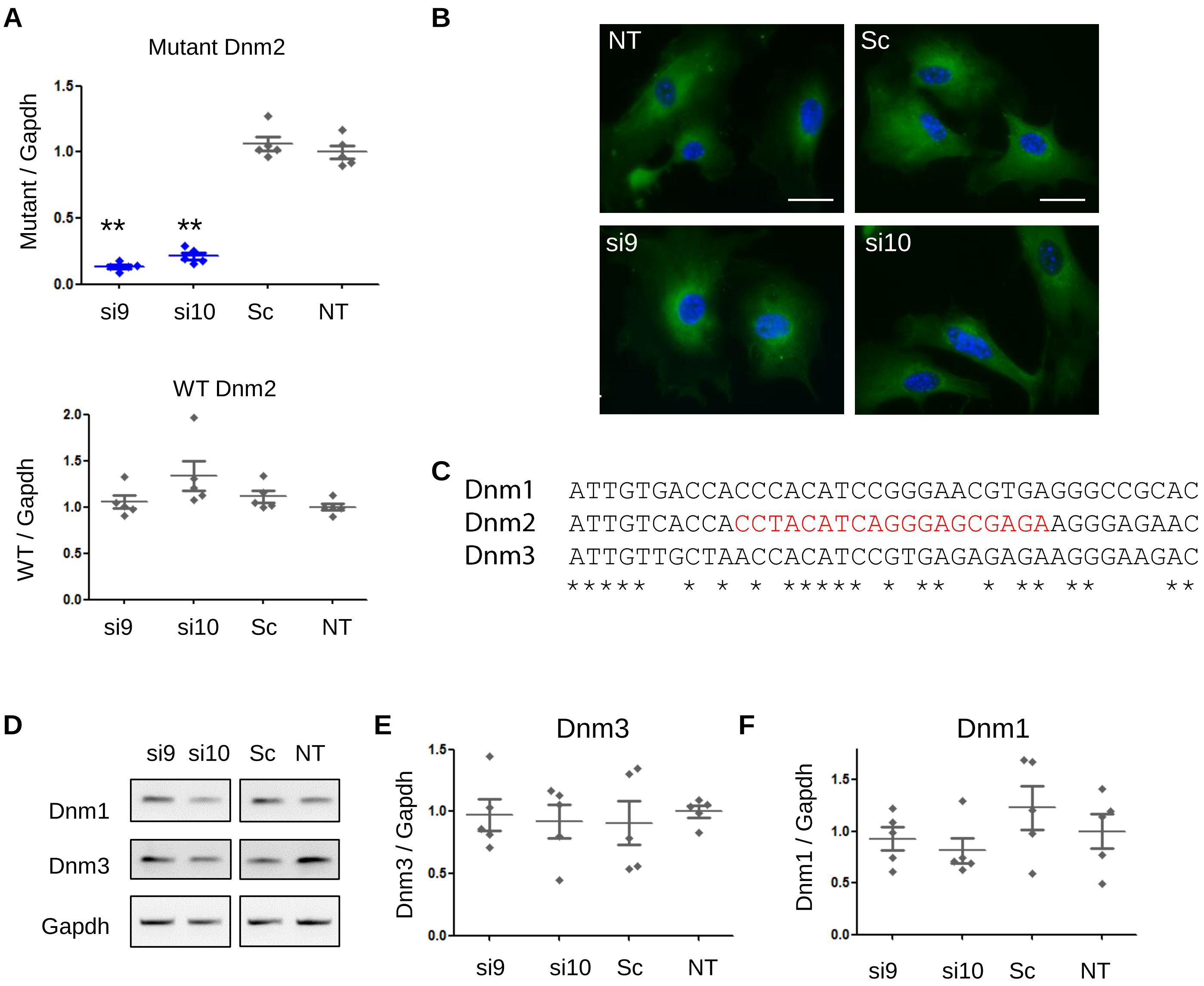

Appendix Figure S2. Effect of the allele-specific si9 and si10 sequences on Dnm1, Dnm2 and Dnm3 expression.

Cells were transfected with siRNAs at 100 nM for 48h.
(**A**) Quantification of the mutated and WT Dnm2 transcripts after RT-PCR and EcoNI digestion and normalization relative to Gapdh expression. Scatter plots bars represent mean ± SEM. ** P<0.01 using a one-tailed Mann-Whitney *U*-test compared to scramble values (n=5).
(**B**) Representative images of Dnm2 immunostaining in non-transfected cells (NT) and in siRNA-treated cells. Scale bars= 10µm.
(**C**) Sequence alignment of the mouse Dnm1, Dnm2 and Dnm3 transcripts in the region targeted by the siRNA. Position of the si9 is indicated in red in the Dnm2 sequence.
(**D**) Agarose gel of RT-PCR products for Dnm1 and Dnm3 in siRNA-transfected and non-transfected (NT) cells. Gapdh was used as loading control.
(**E**) Quantification of Dnm1 transcript relative to Gapdh expression.
(**F**) Quantification of Dnm3 transcript relative to Gapdh expression. In E and F, Scatter plots bars represent mean ± SEM and a statistical analysis using a Mann-Whitney *U*-test compared to scramble values shows no significant differences (n=5).

Appendix Figure S3

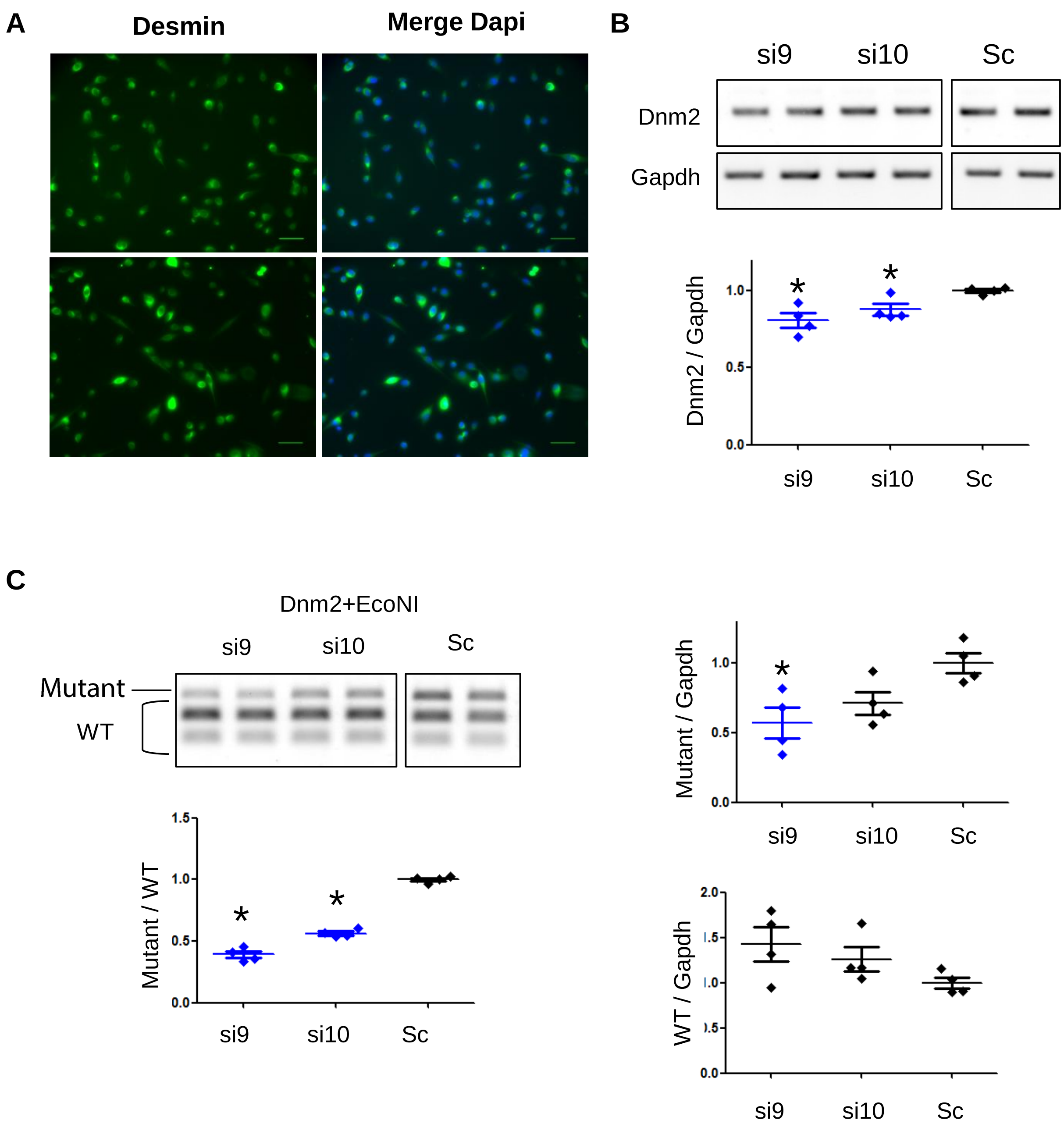

Appendix Figure S3. si9 and si10 are allele-specific siRNAs in Mouse Myoblast

Cells were transfected with siRNAs at 100 nM for 48h.

(**A**) Representative images of Desmin immunostaining in immortalized mouse myoblast. Scale bars= 50µm.

(**B**) *Dnm2* and *Gapdh* semi-quantitative RT-PCR products from siRNA transfected myoblasts and quantification of Dnm2 expression normalized to Gapdh.

(**C**) Quantification of the mutated and WT Dnm2 transcripts after RT-PCR and EcoNI digestion and normalization relative to Gapdh expression. Scatter plots bars represent mean ± SEM. * $P<0.05$ using a one-tailed Mann-Whitney *U*-test compared to scramble values (n=4).

Appendix Figure S4

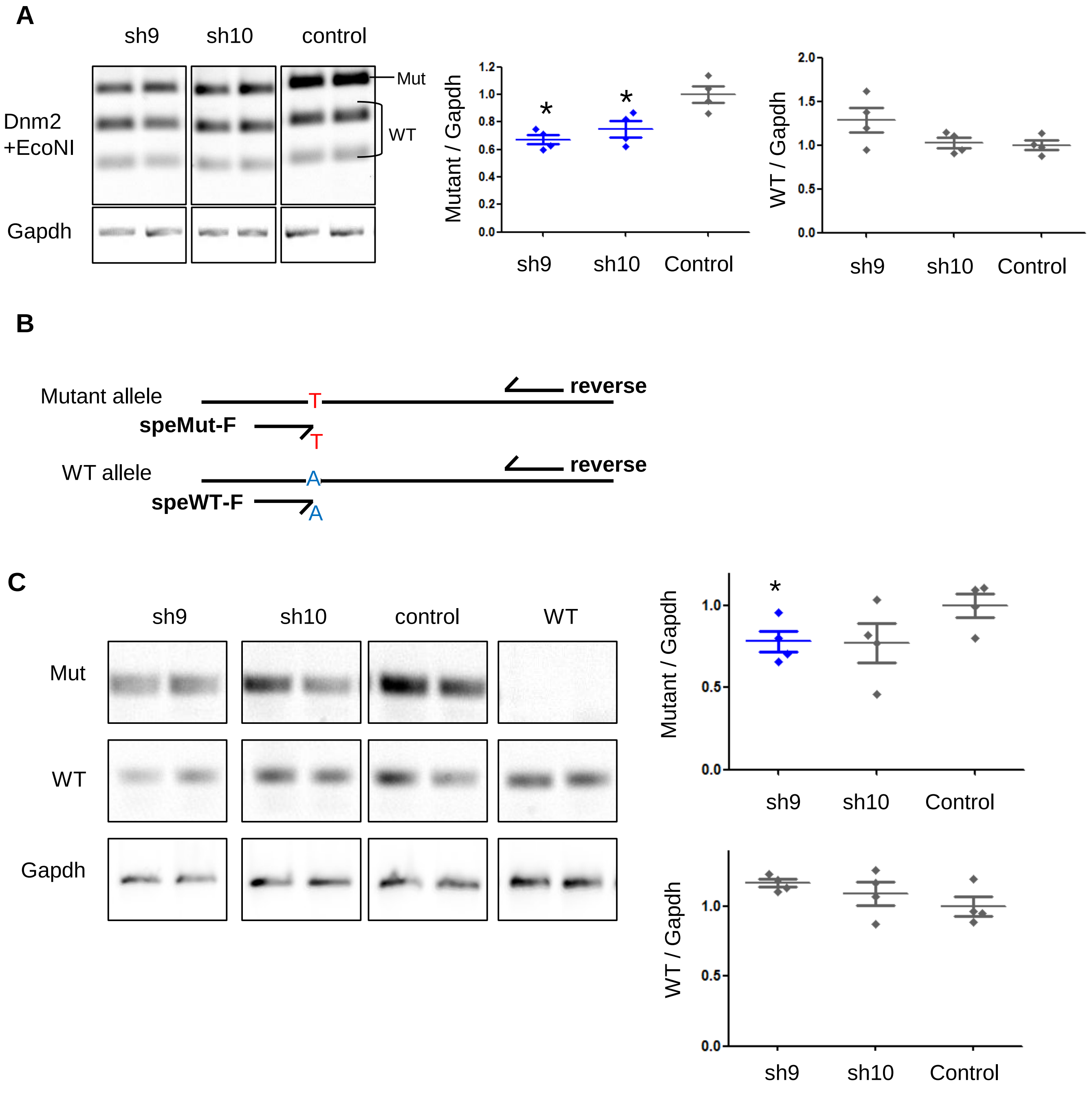

Appendix Figure S4. Expression of WT and mutated Dnm2 mRNAs in AAV-transduced mouse muscles.

(**A**) Agarose gel electrophoresis of RT-PCR products of Gapdh mRNA and Dnm2 mRNA digested by EcoNI. Left panel: quantification of the Mutant and WT Dnm2 amplicons relative to Gapdh. (**B**) Design of RT-PCR assay for specific amplification of either WT or mutant Dnm2 transcripts. For both amplification, the last 3' base of the forward primers (mDnm2speMut-F and mDnm2speWT-F in Appendix Table S1) corresponds to the position of the mutated nucleotide. The reverse primer is common. (**C**) Agarose gel electrophoresis and quantification of allele-specific RT-PCR products of Dnm2 mRNAs relative to Gapdh. Bars of the scatter plots represent mean ± SEM. * $P<0.05$ using a one-tailed Mann-Whitney *U*-test compared to AAV-control values (n=4). Mut: Mutant Dnm2

Appendix Figure S5

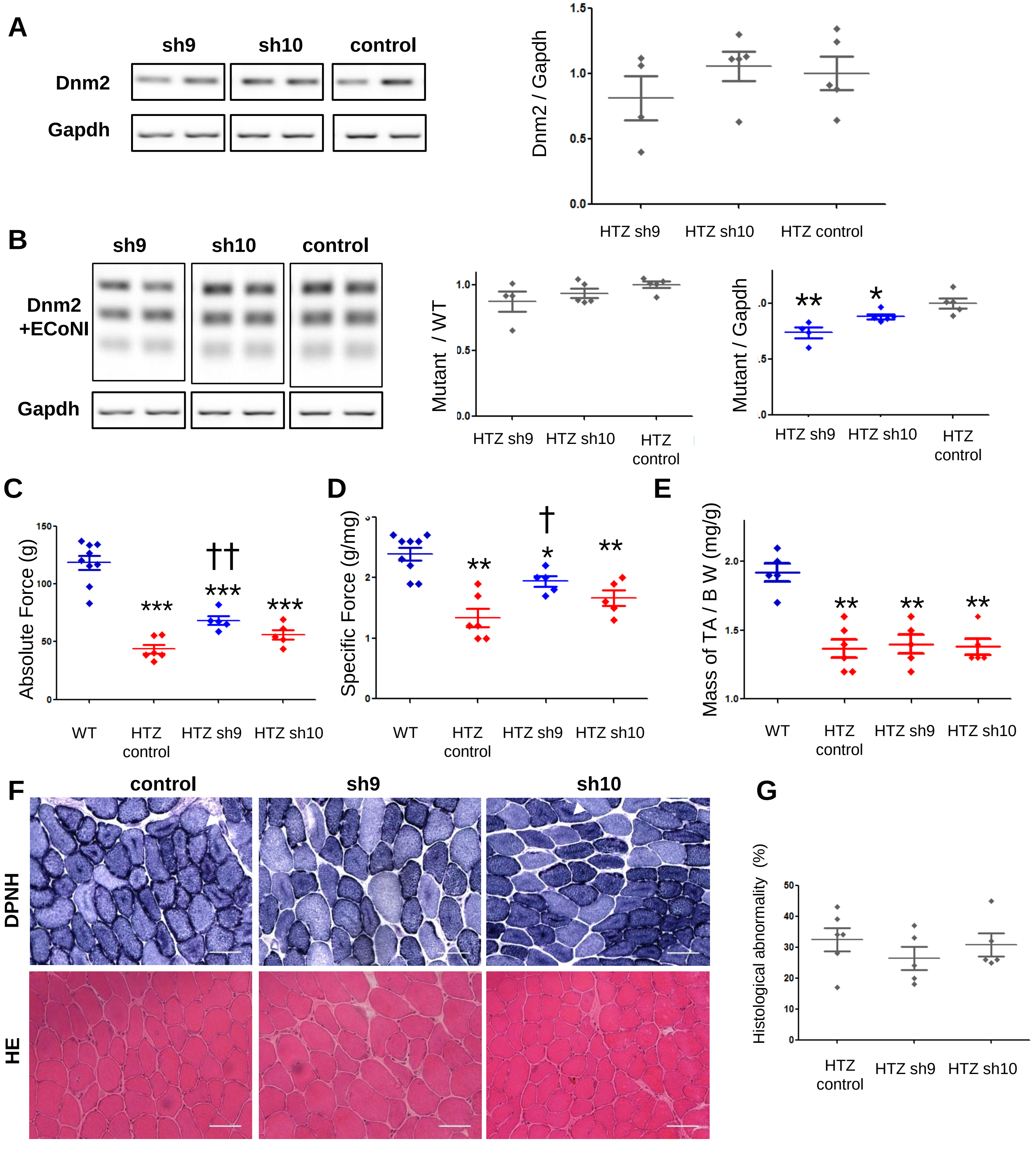

Appendix Figure S5. Effect of 1 month Sh9 treatment in young mice. (**A**) Dnm2 and Gapdh RT-PCR products from TA muscles and quantification of Dnm2 expression normalized to Gapdh. WT muscles were included as control. (n≥4). (**B**) EcoNI digestion profile of the Dnm2 amplicons and quantification of the mutant/WT and mutant/Gapdh ratios. (n≥4) (**C**) Absolute maximal force and (**D**) specific maximal force developed by TA muscles (n≥5). (**E**) Muscle mass in AAV-shRNA injected mice. The TA weights were normalized by the total body weight (mg/g) (n≥5). Scatter plots bars represent mean ± SEM. Statistical analysis was performed using a one-tailed Mann-Whitney U-test. * P<0.05, **P<0.01 and *** P<0.001 compared to WT. † P<0.05, †† P<0.01 compared to AAV-control. BW: Body weight. (**F**) Histochemical staining of TA sections from WT and AAV-injected HTZ mice. HE: hematoxilin eosin staining. DPNH: Reduced diphosphopyridine nucleotide diaphorase staining. Scale bars= 50 µm (**G**) Quantification of histological abnormalities. Scatter plot represents individual percentages of histological abnormalities from heterozygous control or treated mice.(n≥5).

Appendix Figure S6

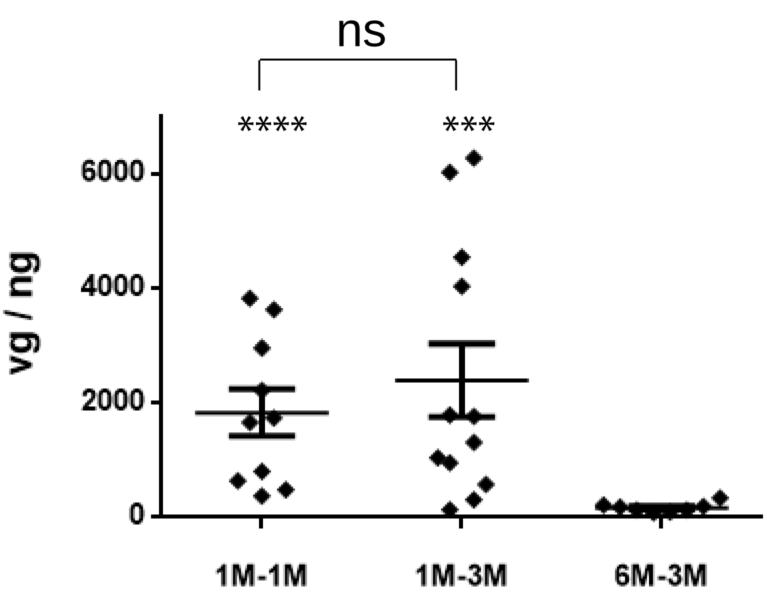

Appendix Figure S6. Quantification of viral genome per ng of DNA. The scatter plot represents individual values of viral genomes per nucleus from the three groups of injected mice, Mean ± SEM are indicated. The viral genome number is plotted per group of mice irrespectively of the AAV type. 1M-1M: mice injected at the age of 1 month for a period of 1 month. 1M-3M: mice injected at the age of 1 month for a period of 3 months; 6M-3M: mice injected at the age of 6 months for a period of 3 months. Statistical analysis was performed using a two-tailed Mann-Whitney U-test ($n \geq 8$). **** $P<0.0001$,*** $P<0.001$ compared to 6M-3M group

Appendix Figure S7

A

```
Mm-si9/10-R465W   ACCTACATCTGGGAGCGAGA
Hs-si9/10-R465W   ACTTACATCTGGGAACGGGA
                  ** *********** ** **
```

B

GAAAAAGCAGGTCGTCAAGCTGAAAGAGCCCTGTCTGAAATGTGTCGACCTGGTTA**TCCAG**
GAGCTAATCAATACAGTTAGGCAGTGTACCAGTAAGCTCAGTTCCTACCCCCGGTTGCGAG
AGGAGACAGAGCGAATCGTCACCACTTACA**TCCGGGA**ACGGGAGGGGAGAACGAAGGACCA
GATTCTTCTGCTGATCGACATTGAGCAGTCCTACATCAACACGAACCATGAGGACTTCATC
GGGTTTGCCAATGCCCAGCAGAGGAGCACGCAGCTGAACAAGAAGAGAGCCATCCCCAAT

C

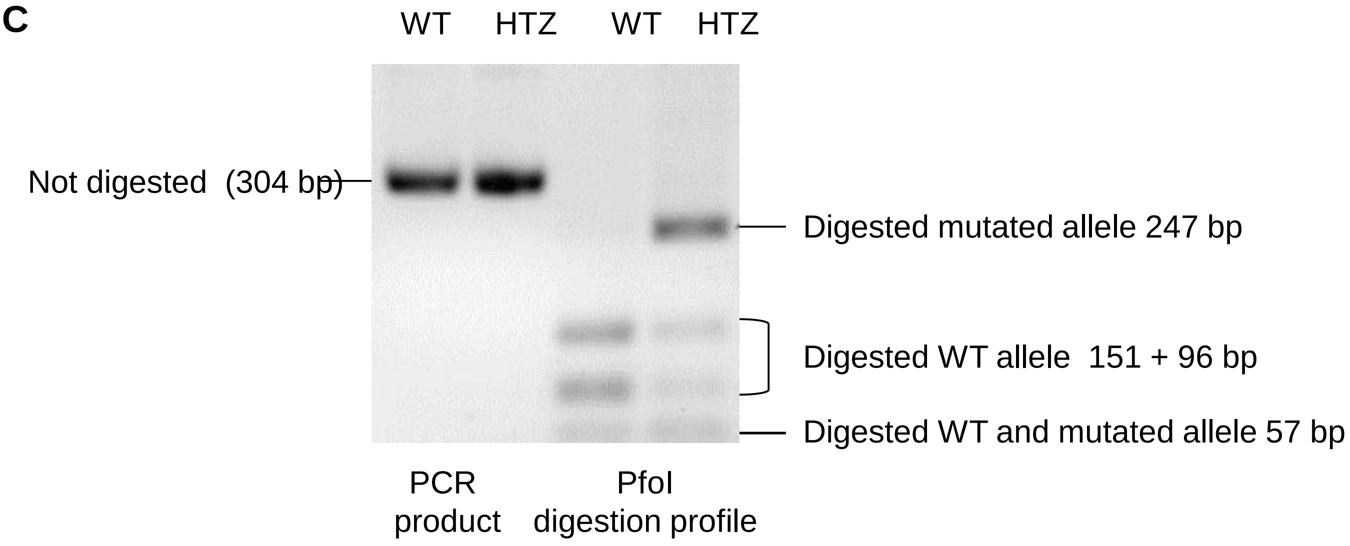

Appendix Figure S7: RT-PCR assay for identification of allele-specific siRNAs in human cells.
(**A**) Sequence alignment of the mouse and human Dnm2 si9/si10 RNAs targeting the R465W mutation in both species. The mutant nucleotide is indicated in red. Mm: mus musculus, Hs: Homo sapiens.
(**B**) Nucleotide sequence of the human DNM2 RT-PCR product in the region of the mutation. Primers are underlined, PfoI sites are in bold and position of the mutation (C>T) in red. Presence of the mutation abolishes the second recognition site for PfoI.
(**C**) Agarose gel electrophoresis of non-digested PCR amplicons (left) or after PfoI digestion (right) for healthy control (WT) and patient (HTZ) cells. PfoI digestion allows discrimination between WT and mutant alleles and quantification of associated-signal with both alleles. The associated-signal for the WT allele is obtained by the sum of the signal for the two bands at 151 and 96 base-pairs. WT sample is used as internal control showing total digestion under these conditions. Length of each band is indicated in base-pairs (bp).

Appendix Figure S8

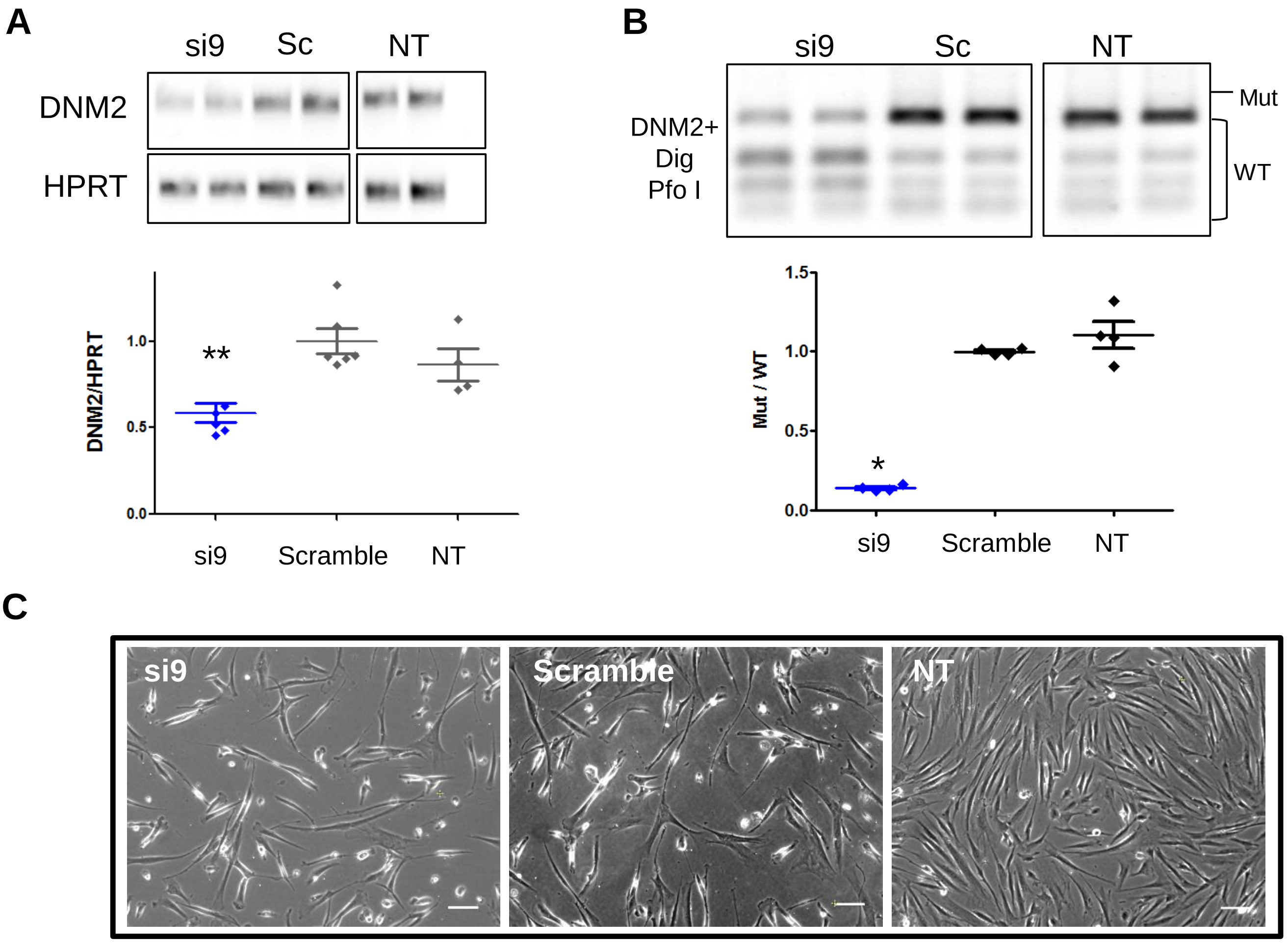

Appendix Figure S8. Allele-specific silencing induced by si9 in human fibroblasts at high doses. (**A**) Agarose gel electrophoresis of DNM2 and HPRT RT-PCR products 48 hours after transfection of siRNAs at 100 nM. Sc: Scramble siRNA. NT: non-transfected cells. Scatter plot represent DNM2 normalized to HPRT (n=5). (**B**) Pfo I digestion profile of the DNM2 amplicons on agarose gel electrophoresis and quantification of the Mutant/WT ratio. The bar of the scatter plot represent mean ± SEM. * P<0.01 ,** P<0.01 using a one tailed Mann-Whitney *U*-test compared to scramble values (n=4). (**C**) Representative pictures of human fibroblasts 48h after transfection with si9 and scramble siRNAs at 100 nM. Scale bars = 50µM.

Appendix Figure S9 A

Results Blastn >>> human si9-RNA 1/1

Sequences producing significant alignments:

Select: All None Selected:0

Alignments Download GenBank Graphics Distance tree of results

	Description	Max score	Total score	Query cover	E value	Ident	Accession
	**Transcripts**						
☐	Homo sapiens dynamin 2 (DNM2), transcript variant 1, mRNA	30.2	30.2	100%	11	95%	NM_001005360.2
☐	Homo sapiens dynamin 2 (DNM2), transcript variant 2, mRNA	30.2	30.2	100%	11	95%	NM_001005361.2
☐	Homo sapiens dynamin 2 (DNM2), transcript variant 4, mRNA	30.2	30.2	100%	11	95%	NM_001005362.2
☐	Homo sapiens dynamin 2 (DNM2), transcript variant 3, mRNA	30.2	30.2	100%	11	95%	NM_004945.3
☐	Homo sapiens dynamin 2 (DNM2), transcript variant 5, mRNA	30.2	30.2	100%	11	95%	NM_001190716.1
☐	PREDICTED: Homo sapiens uncharacterized LOC105377960 (LOC105377960), ncRNA	28.2	28.2	73%	42	100%	XR_942905.1
☐	PREDICTED: Homo sapiens uncharacterized LOC105377960 (LOC105377960), ncRNA	28.2	28.2	73%	42	100%	XR_954924.1
☐	PREDICTED: Homo sapiens uncharacterized LOC105378557 (LOC105378557), ncRNA	28.2	28.2	73%	42	100%	XR_946465.1
☐	PREDICTED: Homo sapiens uncharacterized LOC105378557 (LOC105378557), ncRNA	28.2	28.2	73%	42	100%	XR_960008.1
☐	PREDICTED: Homo sapiens solute carrier family 9, subfamily A (NHE8, cation proton antiporter 8), member 8 (SLC9A8), transcript varian	26.3	26.3	68%	167	100%	XM_011528744.1
☐	PREDICTED: Homo sapiens solute carrier family 9, subfamily A (NHE8, cation proton antiporter 8), member 8 (SLC9A8), transcript varian	26.3	26.3	68%	167	100%	XM_011528743.1
☐	PREDICTED: Homo sapiens solute carrier family 9, subfamily A (NHE8, cation proton antiporter 8), member 8 (SLC9A8), transcript varian	26.3	26.3	68%	167	100%	XM_011528742.1
☐	PREDICTED: Homo sapiens solute carrier family 9, subfamily A (NHE8, cation proton antiporter 8), member 8 (SLC9A8), transcript varian	26.3	26.3	68%	167	100%	XM_011528741.1
☐	PREDICTED: Homo sapiens solute carrier family 9, subfamily A (NHE8, cation proton antiporter 8), member 8 (SLC9A8), transcript varian	26.3	26.3	68%	167	100%	XM_011528739.1
☐	PREDICTED: Homo sapiens solute carrier family 9, subfamily A (NHE8, cation proton antiporter 8), member 8 (SLC9A8), transcript varian	26.3	26.3	68%	167	100%	XM_011528738.1
☐	PREDICTED: Homo sapiens solute carrier family 9, subfamily A (NHE8, cation proton antiporter 8), member 8 (SLC9A8), transcript varian	26.3	26.3	68%	167	100%	XM_011528737.1
☐	PREDICTED: Homo sapiens solute carrier family 9, subfamily A (NHE8, cation proton antiporter 8), member 8 (SLC9A8), transcript varian	26.3	26.3	68%	167	100%	XM_011528736.1
☐	PREDICTED: Homo sapiens uncharacterized LOC105372097 (LOC105372097), ncRNA	26.3	26.3	89%	167	94%	XR_935431.1
☐	PREDICTED: Homo sapiens uncharacterized LOC105371530 (LOC105371530), ncRNA	26.3	26.3	68%	167	100%	XR_934221.1
☐	PREDICTED: Homo sapiens uncharacterized LOC105369432 (LOC105369432), transcript variant X4, ncRNA	26.3	26.3	68%	167	100%	XR_947898.1
☐	PREDICTED: Homo sapiens sorbin and SH3 domain containing 1 (SORBS1), transcript variant X34, mRNA	26.3	26.3	68%	167	100%	XM_011539163.1
☐	PREDICTED: Homo sapiens sorbin and SH3 domain containing 1 (SORBS1), transcript variant X26, mRNA	26.3	26.3	68%	167	100%	XM_011539159.1
☐	PREDICTED: Homo sapiens sorbin and SH3 domain containing 1 (SORBS1), transcript variant X24, mRNA	26.3	26.3	68%	167	100%	XM_011539157.1
☐	PREDICTED: Homo sapiens sorbin and SH3 domain containing 1 (SORBS1), transcript variant X23, mRNA	26.3	26.3	68%	167	100%	XM_011539156.1
☐	PREDICTED: Homo sapiens sorbin and SH3 domain containing 1 (SORBS1), transcript variant X22, mRNA	26.3	26.3	68%	167	100%	XM_011539155.1
☐	PREDICTED: Homo sapiens sorbin and SH3 domain containing 1 (SORBS1), transcript variant X21, mRNA	26.3	26.3	68%	167	100%	XM_011539154.1
☐	PREDICTED: Homo sapiens sorbin and SH3 domain containing 1 (SORBS1), transcript variant X20, mRNA	26.3	26.3	68%	167	100%	XM_011539153.1
☐	PREDICTED: Homo sapiens sorbin and SH3 domain containing 1 (SORBS1), transcript variant X19, mRNA	26.3	26.3	68%	167	100%	XM_011539152.1
☐	PREDICTED: Homo sapiens sorbin and SH3 domain containing 1 (SORBS1), transcript variant X18, mRNA	26.3	26.3	68%	167	100%	XM_011539151.1
☐	PREDICTED: Homo sapiens sorbin and SH3 domain containing 1 (SORBS1), transcript variant X17, mRNA	26.3	26.3	68%	167	100%	XM_011539150.1
☐	PREDICTED: Homo sapiens sorbin and SH3 domain containing 1 (SORBS1), transcript variant X16, mRNA	26.3	26.3	68%	167	100%	XM_011539149.1
☐	PREDICTED: Homo sapiens sorbin and SH3 domain containing 1 (SORBS1), transcript variant X15, mRNA	26.3	26.3	68%	167	100%	XM_011539148.1
☐	PREDICTED: Homo sapiens sorbin and SH3 domain containing 1 (SORBS1), transcript variant X14, mRNA	26.3	26.3	68%	167	100%	XM_011539147.1
☐	PREDICTED: Homo sapiens sorbin and SH3 domain containing 1 (SORBS1), transcript variant X13, mRNA	26.3	26.3	68%	167	100%	XM_011539146.1
☐	PREDICTED: Homo sapiens sorbin and SH3 domain containing 1 (SORBS1), transcript variant X12, mRNA	26.3	26.3	68%	167	100%	XM_011539145.1
☐	PREDICTED: Homo sapiens sorbin and SH3 domain containing 1 (SORBS1), transcript variant X11, mRNA	26.3	26.3	68%	167	100%	XM_011539144.1
☐	PREDICTED: Homo sapiens sorbin and SH3 domain containing 1 (SORBS1), transcript variant X10, mRNA	26.3	26.3	68%	167	100%	XM_011539143.1
☐	PREDICTED: Homo sapiens sorbin and SH3 domain containing 1 (SORBS1), transcript variant X9, mRNA	26.3	26.3	68%	167	100%	XM_011539142.1
☐	PREDICTED: Homo sapiens sorbin and SH3 domain containing 1 (SORBS1), transcript variant X8, mRNA	26.3	26.3	68%	167	100%	XM_011539141.1
☐	PREDICTED: Homo sapiens sorbin and SH3 domain containing 1 (SORBS1), transcript variant X7, mRNA	26.3	26.3	68%	167	100%	XM_011539140.1
☐	PREDICTED: Homo sapiens sorbin and SH3 domain containing 1 (SORBS1), transcript variant X5, mRNA	26.3	26.3	68%	167	100%	XM_011539139.1
☐	PREDICTED: Homo sapiens sorbin and SH3 domain containing 1 (SORBS1), transcript variant X4, mRNA	26.3	26.3	68%	167	100%	XM_011539138.1
☐	PREDICTED: Homo sapiens sorbin and SH3 domain containing 1 (SORBS1), transcript variant X3, mRNA	26.3	26.3	68%	167	100%	XM_011539137.1
☐	PREDICTED: Homo sapiens sorbin and SH3 domain containing 1 (SORBS1), transcript variant X2, mRNA	26.3	26.3	68%	167	100%	XM_011539136.1
☐	PREDICTED: Homo sapiens protein prenyltransferase alpha subunit repeat containing 1 (PTAR1), transcript variant X6, mRNA	26.3	26.3	68%	167	100%	XM_005251981.3
☐	PREDICTED: Homo sapiens protein prenyltransferase alpha subunit repeat containing 1 (PTAR1), transcript variant X5, mRNA	26.3	26.3	68%	167	100%	XM_005251980.3
☐	PREDICTED: Homo sapiens protein prenyltransferase alpha subunit repeat containing 1 (PTAR1), transcript variant X4, mRNA	26.3	26.3	68%	167	100%	XM_011518640.1
☐	PREDICTED: Homo sapiens protein prenyltransferase alpha subunit repeat containing 1 (PTAR1), transcript variant X3, mRNA	26.3	26.3	68%	167	100%	XM_011518639.1
☐	PREDICTED: Homo sapiens protein prenyltransferase alpha subunit repeat containing 1 (PTAR1), transcript variant X2, mRNA	26.3	26.3	68%	167	100%	XM_005251977.3
☐	PREDICTED: Homo sapiens protein prenyltransferase alpha subunit repeat containing 1 (PTAR1), transcript variant X1, mRNA	26.3	26.3	68%	167	100%	XM_005251976.3
☐	PREDICTED: Homo sapiens frizzled class receptor 6 (FZD6), transcript variant X1, misc_RNA	26.3	26.3	68%	167	100%	XR_428385.2
☐	PREDICTED: Homo sapiens uncharacterized LOC105375608 (LOC105375608), ncRNA	26.3	26.3	68%	167	100%	XR_928262.1
☐	PREDICTED: Homo sapiens solute carrier organic anion transporter family, member 6A1 (SLCO6A1), transcript variant X8, mRNA	26.3	26.3	68%	167	100%	XM_011543153.1
☐	PREDICTED: Homo sapiens solute carrier organic anion transporter family, member 6A1 (SLCO6A1), transcript variant X7, mRNA	26.3	26.3	68%	167	100%	XM_011543152.1
	**Genomic sequences** [show first]						

Appendix Figure S9 B

Results Blastn >>> mouse si9-RNA   Page 1/2

Sequences producing significant alignments:

Select: All None Selected:0

Alignments Download GenBank Graphics Distance tree of results

Description	Max score	Total score	Query cover	E value	Ident	Accession
**Transcripts**						
PREDICTED: Mus musculus dynamin 2 (Dnm2), transcript variant X19, misc_RNA	30.2	30.2	100%	5.0	95%	XR_001778793.1
PREDICTED: Mus musculus dynamin 2 (Dnm2), transcript variant X18, mRNA	30.2	30.2	100%	5.0	95%	XM_006509985.3
PREDICTED: Mus musculus dynamin 2 (Dnm2), transcript variant X17, mRNA	30.2	30.2	100%	5.0	95%	XM_006509984.3
PREDICTED: Mus musculus dynamin 2 (Dnm2), transcript variant X15, mRNA	30.2	30.2	100%	5.0	95%	XM_017313129.1
PREDICTED: Mus musculus dynamin 2 (Dnm2), transcript variant X14, mRNA	30.2	30.2	100%	5.0	95%	XM_006509982.2
PREDICTED: Mus musculus dynamin 2 (Dnm2), transcript variant X13, mRNA	30.2	30.2	100%	5.0	95%	XM_006509981.2
PREDICTED: Mus musculus dynamin 2 (Dnm2), transcript variant X12, mRNA	30.2	30.2	100%	5.0	95%	XM_017313128.1
PREDICTED: Mus musculus dynamin 2 (Dnm2), transcript variant X11, mRNA	30.2	30.2	100%	5.0	95%	XM_017313127.1
PREDICTED: Mus musculus dynamin 2 (Dnm2), transcript variant X10, mRNA	30.2	30.2	100%	5.0	95%	XM_017313126.1
PREDICTED: Mus musculus dynamin 2 (Dnm2), transcript variant X9, mRNA	30.2	30.2	100%	5.0	95%	XM_006509980.2
PREDICTED: Mus musculus dynamin 2 (Dnm2), transcript variant X7, mRNA	30.2	30.2	100%	5.0	95%	XM_006509978.2
PREDICTED: Mus musculus dynamin 2 (Dnm2), transcript variant X5, mRNA	30.2	30.2	100%	5.0	95%	XM_017313125.1
PREDICTED: Mus musculus dynamin 2 (Dnm2), transcript variant X4, mRNA	30.2	30.2	100%	5.0	95%	XM_006509976.2
PREDICTED: Mus musculus dynamin 2 (Dnm2), transcript variant X16, mRNA	30.2	30.2	100%	5.0	95%	XM_006509983.1
PREDICTED: Mus musculus dynamin 2 (Dnm2), transcript variant X8, mRNA	30.2	30.2	100%	5.0	95%	XM_006509979.1
PREDICTED: Mus musculus dynamin 2 (Dnm2), transcript variant X6, mRNA	30.2	30.2	100%	5.0	95%	XM_006509977.1
PREDICTED: Mus musculus dynamin 2 (Dnm2), transcript variant X3, mRNA	30.2	30.2	100%	5.0	95%	XM_006509975.1
PREDICTED: Mus musculus dynamin 2 (Dnm2), transcript variant X2, mRNA	30.2	30.2	100%	5.0	95%	XM_006509974.1
PREDICTED: Mus musculus dynamin 2 (Dnm2), transcript variant X1, mRNA	30.2	30.2	100%	5.0	95%	XM_006509973.1
Mus musculus dynamin 2 (Dnm2), transcript variant 4, mRNA	30.2	30.2	100%	5.0	95%	NM_001253894.1
Mus musculus dynamin 2 (Dnm2), transcript variant 3, mRNA	30.2	30.2	100%	5.0	95%	NM_007871.2
Mus musculus dynamin 2 (Dnm2), transcript variant 2, mRNA	30.2	30.2	100%	5.0	95%	NM_001039520.2
Mus musculus dynamin 2 (Dnm2), transcript variant 1, mRNA	30.2	30.2	100%	5.0	95%	NM_001253893.1
PREDICTED: Mus musculus zinc finger protein 599 (Zfp599), transcript variant X1, mRNA	28.2	28.2	73%	20	100%	XM_011242471.2
Mus musculus leucine rich repeat and fibronectin type III domain containing 3 (Lrfn3), mRNA	28.2	28.2	73%	20	100%	NM_175478.2
PREDICTED: Mus musculus predicted gene 16150 (Gm16150), transcript variant X5, ncRNA	28.2	28.2	73%	20	100%	XR_001778895.1
PREDICTED: Mus musculus predicted gene 16150 (Gm16150), transcript variant X4, ncRNA	28.2	28.2	73%	20	100%	XR_001778894.1
PREDICTED: Mus musculus predicted gene 16150 (Gm16150), transcript variant X3, ncRNA	28.2	28.2	73%	20	100%	XR_001778891.1
PREDICTED: Mus musculus predicted gene 16150 (Gm16150), transcript variant X2, ncRNA	28.2	28.2	73%	20	100%	XR_001778890.1
PREDICTED: Mus musculus predicted gene 16150 (Gm16150), transcript variant X1, ncRNA	28.2	28.2	73%	20	100%	XR_865363.2
Mus musculus predicted gene 382 (Gm382), mRNA	28.2	28.2	73%	20	100%	NM_001033241.3
PREDICTED: Mus musculus CLIP associating protein 2 (Clasp2), transcript variant X23, mRNA	26.3	26.3	68%	79	100%	XM_006512397.3
PREDICTED: Mus musculus CLIP associating protein 2 (Clasp2), transcript variant X20, misc_RNA	26.3	26.3	68%	79	100%	XR_379881.3
PREDICTED: Mus musculus CLIP associating protein 2 (Clasp2), transcript variant X15, mRNA	26.3	26.3	68%	79	100%	XM_017313687.1
PREDICTED: Mus musculus CLIP associating protein 2 (Clasp2), transcript variant X14, mRNA	26.3	26.3	68%	79	100%	XM_017313686.1
PREDICTED: Mus musculus CLIP associating protein 2 (Clasp2), transcript variant X13, mRNA	26.3	26.3	68%	79	100%	XM_006512394.3
PREDICTED: Mus musculus CLIP associating protein 2 (Clasp2), transcript variant X12, mRNA	26.3	26.3	68%	79	100%	XM_017313685.1
PREDICTED: Mus musculus CLIP associating protein 2 (Clasp2), transcript variant X11, mRNA	26.3	26.3	68%	79	100%	XM_006512393.3
PREDICTED: Mus musculus CLIP associating protein 2 (Clasp2), transcript variant X10, mRNA	26.3	26.3	68%	79	100%	XM_017313684.1
PREDICTED: Mus musculus CLIP associating protein 2 (Clasp2), transcript variant X9, mRNA	26.3	26.3	68%	79	100%	XM_006512392.3
PREDICTED: Mus musculus CLIP associating protein 2 (Clasp2), transcript variant X8, mRNA	26.3	26.3	68%	79	100%	XM_006512391.3
PREDICTED: Mus musculus CLIP associating protein 2 (Clasp2), transcript variant X7, mRNA	26.3	26.3	68%	79	100%	XM_006512390.3
PREDICTED: Mus musculus CLIP associating protein 2 (Clasp2), transcript variant X6, mRNA	26.3	26.3	68%	79	100%	XM_006512389.3
PREDICTED: Mus musculus CLIP associating protein 2 (Clasp2), transcript variant X5, mRNA	26.3	26.3	68%	79	100%	XM_006512388.3
PREDICTED: Mus musculus CLIP associating protein 2 (Clasp2), transcript variant X4, mRNA	26.3	26.3	68%	79	100%	XM_006512387.3
PREDICTED: Mus musculus CLIP associating protein 2 (Clasp2), transcript variant X3, mRNA	26.3	26.3	68%	79	100%	XM_006512386.3
PREDICTED: Mus musculus CLIP associating protein 2 (Clasp2), transcript variant X2, mRNA	26.3	26.3	68%	79	100%	XM_006512385.3
PREDICTED: Mus musculus CLIP associating protein 2 (Clasp2), transcript variant X1, mRNA	26.3	26.3	68%	79	100%	XM_006512384.3
PREDICTED: Mus musculus cysteine-serine-rich nuclear protein 1 (Csrnp1), transcript variant X3, mRNA	26.3	26.3	68%	79	100%	XM_006512038.2
PREDICTED: Mus musculus myosin VA (Myo5a), transcript variant X7, mRNA	26.3	26.3	68%	79	100%	XM_006510834.3
PREDICTED: Mus musculus myosin VA (Myo5a), transcript variant X6, mRNA	26.3	26.3	68%	79	100%	XM_006510832.3
PREDICTED: Mus musculus myosin VA (Myo5a), transcript variant X5, mRNA	26.3	26.3	68%	79	100%	XM_006510831.3
PREDICTED: Mus musculus myosin VA (Myo5a), transcript variant X4, mRNA	26.3	26.3	68%	79	100%	XM_006510830.3
PREDICTED: Mus musculus myosin VA (Myo5a), transcript variant X3, mRNA	26.3	26.3	68%	79	100%	XM_006510829.3
PREDICTED: Mus musculus myosin VA (Myo5a), transcript variant X2, mRNA	26.3	26.3	68%	79	100%	XM_006510828.3
PREDICTED: Mus musculus myosin VA (Myo5a), transcript variant X1, mRNA	26.3	26.3	68%	79	100%	XM_006510827.3
PREDICTED: Mus musculus xylulokinase homolog (H. influenzae) (Xylb), transcript variant X3, mRNA	26.3	26.3	68%	79	100%	XM_017313063.1
PREDICTED: Mus musculus xylulokinase homolog (H. influenzae) (Xylb), transcript variant X2, mRNA	26.3	26.3	68%	79	100%	XM_006511908.3
PREDICTED: Mus musculus xylulokinase homolog (H. influenzae) (Xylb), transcript variant X1, mRNA	26.3	26.3	68%	79	100%	XM_011242927.2
PREDICTED: Mus musculus paired box 5 (Pax5), transcript variant X2, mRNA	26.3	26.3	68%	79	100%	XM_006537674.3
PREDICTED: Mus musculus paired box 5 (Pax5), transcript variant X1, mRNA	26.3	26.3	68%	79	100%	XM_006537673.3
PREDICTED: Mus musculus zinc finger protein 516 (Zfp516), transcript variant X5, mRNA	26.3	26.3	68%	79	100%	XM_006526499.3
PREDICTED: Mus musculus uncharacterized LOC108168287 (LOC108168287), ncRNA	26.3	26.3	68%	79	100%	XR_001781926.1
PREDICTED: Mus musculus caspase recruitment domain family, member 6 (Card6), transcript variant X3, mRNA	26.3	26.3	68%	79	100%	XM_011245331.2
PREDICTED: Mus musculus caspase recruitment domain family, member 6 (Card6), transcript variant X2, mRNA	26.3	26.3	68%	79	100%	XM_006519989.3
PREDICTED: Mus musculus caspase recruitment domain family, member 6 (Card6), transcript variant X1, mRNA	26.3	26.3	68%	79	100%	XM_006519987.3
PREDICTED: Mus musculus 5'-nucleotidase domain containing 2 (Nt5dc2), transcript variant X1, mRNA	26.3	26.3	68%	79	100%	XM_006519507.2
PREDICTED: Mus musculus predicted gene 12059 (Gm12059), transcript variant X2, misc_RNA	26.3	26.3	68%	79	100%	XR_381097.3
PREDICTED: Mus musculus dynein, axonemal, intermediate chain 2 (Dnaic2), transcript variant X3, mRNA	26.3	26.3	68%	79	100%	XM_006533662.3
PREDICTED: Mus musculus syntrophin, gamma 1 (Sntg1), transcript variant X6, mRNA	26.3	26.3	68%	79	100%	XM_017312324.1
PREDICTED: Mus musculus syntrophin, gamma 1 (Sntg1), transcript variant X2, mRNA	26.3	26.3	68%	79	100%	XM_017312320.1
PREDICTED: Mus musculus dynein, axonemal, intermediate chain 2 (Dnaic2), transcript variant X4, mRNA	26.3	26.3	68%	79	100%	XM_006533663.2
PREDICTED: Mus musculus dynein, axonemal, intermediate chain 2 (Dnaic2), transcript variant X1, mRNA	26.3	26.3	68%	79	100%	XM_006533660.2

Appendix Figure S9 B

Results Blastn >>> mouse si9-RNA   Page 2/2

PREDICTED: Mus musculus dynein, axonemal, intermediate chain 2 (Dnaic2), transcript variant X4, mRNA	26.3	26.3	68%	79	100%	XM_006533663.2
PREDICTED: Mus musculus dynein, axonemal, intermediate chain 2 (Dnaic2), transcript variant X1, mRNA	26.3	26.3	68%	79	100%	XM_006533660.2
PREDICTED: Mus musculus predicted gene 12059 (Gm12059), transcript variant X1, misc_RNA	26.3	26.3	68%	79	100%	XR_872307.1
PREDICTED: Mus musculus formiminotransferase cyclodeaminase (Ftcd), transcript variant X2, mRNA	26.3	26.3	68%	79	100%	XM_006513216.2
PREDICTED: Mus musculus formiminotransferase cyclodeaminase (Ftcd), transcript variant X1, mRNA	26.3	26.3	68%	79	100%	XM_006513215.2
Mus musculus CD83 antigen (Cd83), transcript variant 1, mRNA	26.3	26.3	68%	79	100%	NM_009856.3
Mus musculus CD83 antigen (Cd83), transcript variant 2, mRNA	26.3	26.3	68%	79	100%	NM_001289915.1
PREDICTED: Mus musculus dynein, axonemal, intermediate chain 2 (Dnaic2), transcript variant X2, mRNA	26.3	26.3	68%	79	100%	XM_006533661.1
Mus musculus CLIP associating protein 2 (Clasp2), transcript variant 4, mRNA	26.3	26.3	68%	79	100%	NM_001286599.1
Mus musculus RIKEN cDNA A630023P12 gene (A630023P12Rik), long non-coding RNA	26.3	26.3	68%	79	100%	NR_102290.1

Genomic sequences [show first]

Appendix Figure S10

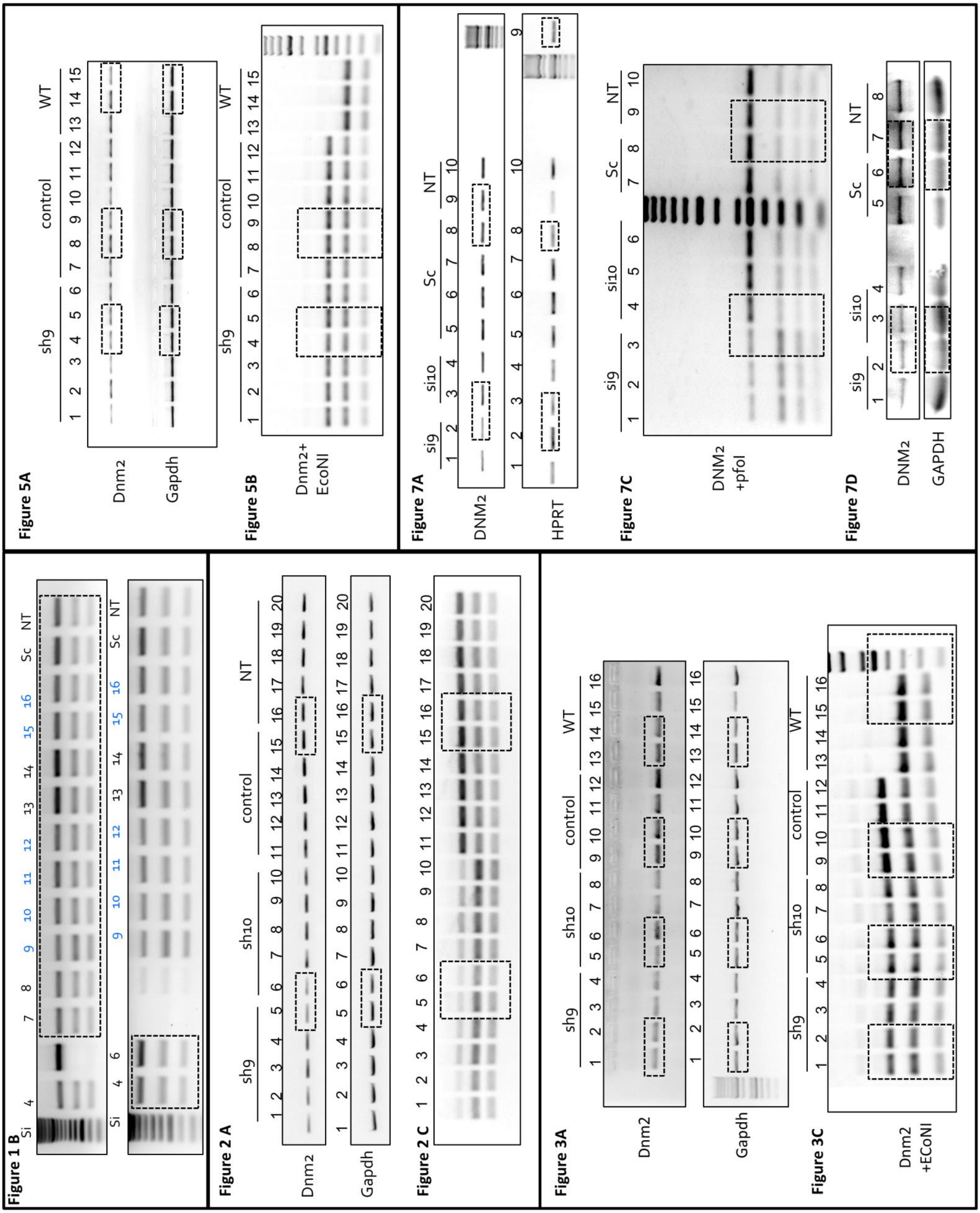

Appendix Figure S10. *Uncropped gels* and western blots from main Figures. The black boxes indicate the cropped regions. For HPRT in Figure 7A sample 9 has been loaded twice due to a loading issue the first time. The second load (square 9 after the molecular size marker) has been used for quantification

Appendix Figure S11

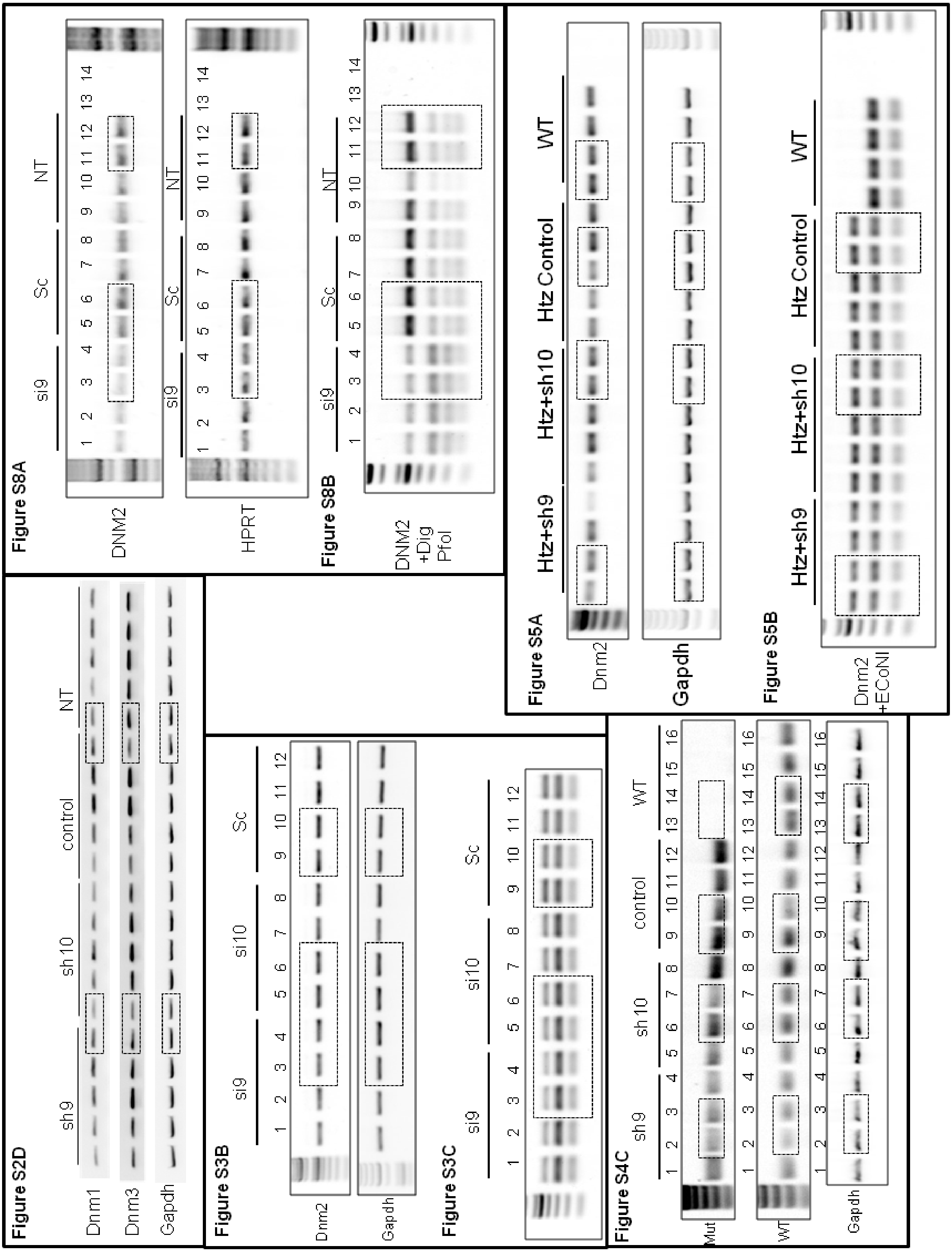

Appendix Figure S11. ***Uncropped gels*** **and western blots from appendix Figures** The black boxes indicate the cropped regions.

Target	Name	siRNA sequences	
Mouse Dnm2 p.R465W, c.1393 T	si9	Sense	5' CCUACAUC**U**GGGAGCGAGA-dTdT 3'
		Antisense	3' dTdT-GGAUGUAG**A**CCCUCGCUCU 5'
	si10	Sense	5' ACCUACAUC**U**GGGAGCGAG-dTdT 3'
		Antisense	3' dTdT-UGGAUGUAG**A**CCCUCGCUC 5'
Human Dnm2 p.R465W, c.1393 T	si9	Sense	5' CUUACAUC**U**GGGAACGGGA-dTdT 3'
		Antisense	3'dTdT-GAAUGUAG**A**CCCUUGCCCU 5'
	si10	Sense	5' ACUUACAUC**U**GGGAACGGG-dTdT 3'
		Antisense	3 ' dTdT-UGAAUGUAG**A**CCCUUGCCC 5'

Appendix Table S1: siRNA molecules targeting Dnm2 mutant mRNA in this study.

Target		Sequence (5'-3')	Application
**mGapdh**	F	ACCACAGTCCATGCCATCAC	Semi-quantitative analysis in mouse
	R	TCCACCACCCTGTTGCTGTA	
**hHPRT**	F	ACCCCACGAAGTGTTGGATA	Semi-quantitative analysis in human cells
	R	AAGCAGATGGCCACAGAACT	
**mDnm2**	F	GGTGGTCAAGCTGAAAGAG	Semi-quantitative analysis in MEF and EcoNI digestion profile
	R	GCTGTCAGCACGAACCAGTA	
**mDnm1**	F	AGATGGAGCGAATTGTGACC	Semi-quantitative analysis in mouse
	R	GAATGACCTGGTTCCCTGAA	
**mDnm3**	F	ATGCTCCGAATGTACCAAGC	Semi-quantitative analysis in mouse
	R	GAGGGGAGCACTTATCGTCA	
**mDnm2b**	F	GGTGGTCAAGCTGAAAGAGC	Semi-quantitative analysis in mouse muscle and EcoNI digestion profile
	R	GGTTTGTGTTGATGTACGACTGC	
**mDnm2speMut**	F	AATTGTCACCACCTACATCT	Specific amplification of the mutant Dnm2 in mouse
	R	GGTTTGTGTTGATGTACGACTGC	
**mDnm2speWT**	F	AATTGTCACCACCTACATCA	Specific amplification of the WT Dnm2 in mouse
	R	GGTTTGTGTTGATGTACGACTGC	
**mOas1**	F	CAGCCTTTGATGTCCTGGGT	Semi-quantitative analysis in mouse
	R	AATGGCTTCCCCAGCTTCT	
**mStat1**	F	GGATCGCTTGCCCAACTCT	Semi-quantitative analysis in mouse
	R	AATGGCTTCCCCAGCTTCT	
**mDnm2g**	F	CTGCGAGAGGAGACCGAGC	Genotyping (knock-in mice)
	R	GCTGAGCACTGGAGAGTGTATGG	
**AAV**	F	CTCCATCACTAGGGGTTCCTTG	Viral genome quantification
	R	GTAGATAAGTAGCATGGC	
	P	TAGTTAATGATTAACCC	
**Titin**	F	AAAACGAGCAGTGACGTGAGC	Viral genome quantification
	R	TTCAGTCATGCTGCTAGCGC	
	P	TGCACGGAAGCGTCTCGTCTCAGTC	
**hSCL9A8**	F	TATGCCCTGAGCCTACACCT	Semi-quantitative analysis in human
	R	CTCCACAGTGTTGCCCATCT	
**hDNM2**	F	GAAAAAGCAGGTCGTCAAGC	Semi-quantitative analysis in human cells and digestion profile
	R	ATTGGGGATGGCTCTCTT	

Appendix Table S2: Primers used in this study. F: Forward. R: Reverse. P: TaqMan probe. mGapdh: Mouse Glyceraldehyde 3-phosphate dehydrogenase. hHPRT: Human hypoxanthine guanine phosphoribosyl transferase. mDnm2: Mouse Dynamin 2. mDnm1: Mouse Dynamin 1. mDnm3: Mouse Dynamin 3. mDnm2speMut: Mouse Dynamin 2 (mutant-specific primers). mDnm2speWT: Mouse Dynamin 2 (wild-type-specific primers). mOas1: Mouse Oligoadenylate synthetase 1. mStat1: Mouse Signal transducer and activator of transcription 1. mDnm2g: Mouse Dynamin2 (genomic DNA). AAV: Adeno-associated virus sequence. hSCL9A8: Human Solute carrier family 9 member A8. hDNM2: Human Dynamin 2.

Table S3: P values main figures

Figure 1B		
**Mann-whitney**	**P value (two-tailed)**	
si4 vs Sc	0.6857	
si6 vs Sc	0.6286	
si7 vs Sc	0.0571	
si8 vs Sc	0.3429	
si9 vs Sc	0.0286	*
si10 vs Sc	0.0286	*
si11 vs Sc	0.0286	*
si12 vs Sc	0.0286	*
s13 vs Sc	0.1143	
si14 vs Sc	1	
si15 vs Sc	0.0286	*
s16 vs Sc	0.0286	*

Figure 2A		
**Mann-whitney**	**P value (one-tailed)**	
si 9 vs Sc	0.004	**
si 10 vs Sc	0.004	**

Figure 2C		
**Mann-whitney**	**P value (one-tailed)**	
si 9 vs Sc	0.004	**
si 10 vs Sc	0.004	**

Figure 2D		
**Mann-whitney**	**P value (one-tailed)**	
si 9 vs Sc	0.004	**
si 10 vs Sc	0.004	**

Figure 3A - Dnm2/Gapdh		
**Mann-whitney**	**P value (one-tailed)**	
sh 9 vs Control	0.0143	*
sh 10 vs Control	0.0286	*

Figure 3C - Mutant/WT		
**Mann-whitney**	**P value (one-tailed)**	
sh 9 vs Control	0.0143	*
sh 10 vs Control	0.0143	*

Figure 4A - Mass of TA		
**Mann-whitney**	**P value (one-tailed)**	
HTZ control vs WT	0.0004	***

sh 9 vs Control	0.0006	***
sh 10 vs Control	0.0031	**
Sh9 vs WT	0.2552	
Sh10 vs WT	0.0025	**

**Figure 4C- Histological abnormalities**		
**Mann-whitney**	**P value (one-tailed)**	
sh 9 vs Control	0.0286	*
sh 10 vs Control	0.1714	

**Figure 4E - Absolute Force**		
**Mann-whitney**	**P value (one-tailed)**	
HTZ control vs WT	< 0.0001	***
sh 9 vs Control	< 0.0001	***
sh 10 vs Control	0.0043	**
Sh9 vs WT	0.1172	
Sh10 vs WT	< 0.0001	***

**Figure 4F - Specific Force**		
**Mann-whitney**	**P value (one-tailed)**	
HTZ control vs WT	0.0088	**
sh 9 vs Control	0.0152	*
sh 10 vs Control	0.2452	
Sh9 vs WT	0.4788	
Sh10 vs WT	0.0364	*

**Figure 5A - Dnm2/Gapdh**		
**Mann-whitney**	**P value (one-tailed)**	
sh9 vs Control	0.066	
sh9 vs WT	0.1762	

**Figure 5B -Mutant/WT**		
**Mann-whitney**	**P value (one-tailed)**	
sh9 vs Control	0.0011	**
**Figure 5C- Histological abnormalities**		
**Mann-whitney**	**P value (one-tailed)**	
sh 9 vs Control	0.2343	

**Figure 5E- Mass of TA/BW**		
**Mann-whitney**	**P value (one-tailed)**	
sh9 vs Control	0.4643	**
HTZ control vs WT	0.0013	**

**Figure 5F - Absolute Force**		

Mann-whitney	P value (one-tailed)	
sh9 vs Control	0.0089	**
sh9 vs WT	0.0005	***
HTZ control vs WT	0.0004	***

Figure 5G - specific force		
**Mann-whitney**	**P value (one-tailed)**	
sh9 vs Control	0.0083	**
sh9 vs WT	0.0335	*
HTZ control vs WT	0.0018	**

Figure 6A -Young mice		
**Mann-whitney Oas1**	**P value (two-tailed)**	
Oas1-sh9 vs Oas1 Control	1	
Oas1-sh10 vs Oas1 Control	0.8857	
Oas1-sh9 vs Oas1 WT	0.6857	
Oas1-sh10 vs Oas1 WT	0.3429	
**Mann-whitney Stat 1**	**P value (two-tailed)**	
Stat1-sh9 vs Stat1 Control	0.6857	
Stat1-sh10 vs Stat1 Control	0.8857	
Stat1-sh9 vs Stat1 WT	1	
Stat1-sh10 vs Stat1 WT	0.8857	

Figure 6A -Old mice		
**Mann-whitney Oas1**	**P value (two-tailed)**	
Oas1-sh9 vs Oas1 Control	0.3939	
Oas1-sh9 vs Oas1 WT	0.2619	
**Mann-whitney Stat 1**	**P value (two-tailed)**	
Stat1-sh9 vs Stat1 Control	0.3095	
Stat1-sh9 vs Stat1 WT	1	

Figure 6B		
**Mann-whitney**	**P value (two-tailed)**	
sh9 (young) vs sh10 (young)	0.6571	
sh10 (young) vs control (young)	0.2	
sh9 (young) vs control (young)	0.2	
sh9 (old) vs control (old)	0.06	
sh9 (young) vs sh9 (old)	0.0286	*
control (young) vs control (old	0.0286	*

Figure 6C		
**Mann-whitney**	**P value (two-tailed)**	
1 month vs 6months (6.10^10vg)	0.0047	**

1 month vs 6months (6.10^11vg)	0.1778	
6months (6.10^10vg) vs 6months (6.10^11vg)	0.0152	*

**Figure 7A - DNM2/HPRT**		
**Mann-whitney**	**P value (one-tailed)**	
si 9 vs Sc	0.0013	**
si 10 vs Sc	0.5	
Sc vs NT	0.4381	

**Figure 7C - Mutant/WT**		
**Mann-whitney**	**P value (one-tailed)**	
si 9 vs Sc	0.0097	**
si 10 vs Sc	0.0159	*

**Figure 7D - DNM2/GAPDH Protein**		
**Mann-whitney**	**P value (one-tailed)**	
si 9 vs Sc	0.0006	***
si 10 vs Sc	0.0012	**

**Figure 7E - SLC9A8/HPRT**		
**Mann-whitney**	**P value (two-tailed)**	
si 9 vs Sc	0.8857	
si 9 vs NT	0.4857	

**Figure 7F - Transferrin Uptake**		
**Mann-whitney**	**P value (two-tailed)**	
CNM Sc vs CNM-si9	< 0.0001	***
CNM Sc vs C1-sc	< 0.0001	***
CNM Sc vs C2-sc	< 0.0001	***

Table S4: P values Supplemental Figures

**Figure S2A -Mutant/Gapdh**		
**Mann-whitney**	**P value (one-tailed)**	
si 9 vs Sc	0.004	**
si 10 vs Sc	0.004	**

**Figure S2A -WT/Gapdh**		
**Mann-whitney**	**P value (two-tailed)**	
si9 vs Sc	0.4206	
si10 vs Sc	0.1508	

**Figure S2E -Dnm3/Gapdh**		
**Mann-whitney**	**P value (two-tailed)**	
si9 vs Sc	0.5476	
si10 vs Sc	1	

**Figure S2F - Dnm1/Gapdh**		
**Mann-whitney**	**P value (two-tailed)**	
si9 vs Sc	0.5476	
si10 vs Sc	0.3095	

**Figure S3B - Dnm2/Gapdh**		
**Mann-whitney**	**P value (one-tailed)**	
**si9 vs Sc**	**0.0143**	*
**si10 vs Sc**	**0.0286**	*

**Figure S3C - Mutated Dnm2**		
**Mann-whitney**	**P value (one-tailed)**	
**si9 vs Sc**	**0.0143**	*
**si10 vs Sc**	**0.0143**	*

**Figure S3C - Mutant/Gapdh**		
**Mann-whitney**	**P value (one-tailed)**	
**si9 vs Sc**	**0.0143**	*
**si10 vs Sc**	**0.0571**	

**Figure S4A - Mutated Dnm2**		
**Mann-whitney**	**P value (one-tailed)**	
sh9 vs control	0.0143	*
sh10 vs control	0.0286	*

**Figure S4A - WT Dnm2**		
**Mann-whitney**	**P value (two-tailed)**	

sh9 vs Sc	0.2	
sh10 vs Sc	0.8857	

Figure S4C - Mutant/Gapdh		
**Mann-whitney**	**P value (one-tailed)**	
sh9 vs control	0.0286	*
sh10 vs control	0.1	

Figure S4C - WT/Gapdh		
**Mann-whitney**	**P value (two-tailed)**	
si9 vs Sc	0.2	
si10 vs Sc	0.6857	

Figure S5A -Dnm2/Gapdh		
**Mann-whitney**	**P value (one-tailed)**	
sh9 vs control	0.2778	
sh10 vs control	0.5	
sh9 vs NT	0.0143	

Figure S5B - Mut/WT		
**Mann-whitney**	**P value (one-tailed)**	
sh9 vs control	0.0952	
sh10 vs control	0.0754	
**Figure S5B - Mut/Gapdh**	**P value**	
**Mann-whitney**	**P value (one-tailed)**	
sh9 vs control	0.0079	**
sh10 vs control	0.0159	*

Figure S5C - Absolute Force		
**Mann-whitney**	**P value (one-tailed)**	
HTZ control vs WT	0.0002	***
sh 9 vs Control	0.0022	**
sh 10 vs Control	0.0628	
Sh9 vs WT	0.0005	***
Sh10 vs WT	0.0002	***

Figure S5D - Specific Force		
**Mann-whitney**	**P value (one-tailed)**	
HTZ control vs WT	0.0013	**
sh 9 vs Control	0.0139	*

sh 10 vs Control	0.0716	
Sh9 vs WT	0.0192	*
Sh10 vs WT	0.0055	**

**Figure S5E - Mass of TA**		
**Mann-whitney**	**P value (one-tailed)**	
HTZ control vs WT	0.004	**
sh 9 vs Control	0.3901	
sh 10 vs Control	0.4255	
Sh9 vs WT	0.004	**
Sh10 vs WT	0.0056	**

**Figure S5G - Histological Abnormalities**		
**Mann-whitney**	**P value (one-tailed)**	
sh 10 vs Control	0.2609	
sh 9 vs Control	0.1571	

**Figure S6**		
**Mann-whitney**	**P value (two-tailed)**	
1M-1M vs 1M-3M	**0.7060**	
1M-1M vs 6M-3M	**< 0.0001**	****
1M-3M vs 6M-3M	**0.0003**	***

**Figure S8A**		
**Mann-whitney**	**P value (one-tailed)**	
si 9 vs Sc	0.0011	**

**Figure S8B**		
**Mann-whitney**	**P value (one-tailed)**	
	0.0143	*
